# Recent advancements in new tracers from first-in-human studies

**DOI:** 10.1007/s12149-024-01979-5

**Published:** 2024-09-26

**Authors:** Yuji Nakamoto, Yoshitaka Inui, Masatoshi Hotta, Hiroshi Wakabayashi, Hirofumi Hanaoka

**Affiliations:** 1 Department of Diagnostic Imaging and Nuclear Medicine, Graduate School of Medicine, Kyoto University, 54 Shogoinkawahara-Cho, Sakyo-Ku, Kyoto, 606-8507 Japan; 2 Department of Radiology, Fujita Health University, 1-98 Dengakugakubo, Kutsukake-Cho, Toyoake, Aichi 470-1192 Japan; 3 Department of Radiology and Nuclear Medicine, National Center for Global Health and Medicine, 1-21-1 Toyama Shinjuku-Ku, Tokyo, 162-8655 Japan; 4 Department of Nuclear Medicine, Kanazawa University Hospital, 13-1 Takaramachi, Kanazawa, Ishikawa 920-8641 Japan; 5 Division of Fundamental Technology Development, Near InfraRed Photo-ImmunoTherapy Research Institute at Kansai Medical University, 2-5-1, Shin-Machi, Hirakata, 573-1010 Japan

**Keywords:** First-in-human, PET, Tracers, Radiopharmaceutical, Neurology, Oncology

## Abstract

Recent advancements in the development of positron emission tomography (PET) tracers have significantly enhanced our ability to image neuroinflammatory processes and neurotransmitter systems, which are vital for understanding and treating neurodegenerative and psychiatric disorders. Similarly, innovative tracers in oncology provide detailed images of the metabolic and molecular characteristics of tumors, which are crucial for tailoring targeted therapies and monitoring responses, including radiotherapy. Notable advancements include programmed death ligand 1 (PD-L1)-targeting agents for lung cancer, prostate-specific membrane antigen-based tracers for prostate cancer, chemokine receptor-targeting agents for hematological malignancies, human epidermal growth factor receptor 2 (HER2)-targeting tracers for various cancers, Claudin 18 based tracers for epithelial tumors, glutamine tracers for colorectal cancer, and ascorbic acid analogs for assessing cancer metabolism and therapy efficacy. Additionally, novel tracers have been developed for non-neurological and non-oncological applications, including adrenal imaging, amyloidosis, and human immunodeficiency virus (HIV) infection. This overview focuses on the newly developed tracers, particularly those used in neurology and oncology.

## Introduction

Several positron emission tomography (PET) tracers have been developed in recent years. In the 1990s, the usefulness of [^18^F]-fluorodeoxyglucose (FDG) for malignant tumors was demonstrated, and it is now routinely used in several facilities. Beyond [^18^F]-FDG, new tracers that target specific receptors and molecules have significantly improved the cancer diagnosis and treatment, and are relevant for targeted therapy adaptation and response monitoring, including radiotherapy. PET examinations of somatostatin receptors using [^68^Ga]-DOTATATE or [^68^Ga]-DOTATOC are widely accepted worldwide, and PSMA-based PET scans are indispensable for determining therapeutic strategies for prostate cancer in clinical settings. Fibroblast activation protein inhibitor-based PET has recently gained popularity and is expected to be widely applied [1]. Other than oncology, a PET examination with amyloid-β is being covered by insurance in Japan. Nuclear medicine imaging methods that capture specific information depend on advances in radiopharmaceuticals. This review article introduces new tracers recently reported in first-in-human studies, mainly in neurology and oncology.

## Innovative tracers in neurology

In the central nervous system (CNS), endocannabinoid signaling is associated with the synaptic plasticity modulation of other neuronal transmitters and cytokine release from microglia. It has been implicated in various neurological and psychiatric disorders. Monoacylglycerol lipase (MAGL) regulates cannabinoid neurotransmission and the pro-inflammatory arachidonic acid pathway by degrading endocannabinoids. MAGL inhibition decreases arachidonic acid levels, resulting in anti-inflammatory and neuroprotective effects in the brain. Thus, MAGL could be a promising target for drug development in neuropsychiatric disorders wherein the endocannabinoid system and neuroinflammation are implicated. Takahata et al. developed [^18^F]-T-401 as a PET ligand for assessing MAGL and provided the first demonstration of the in vivo visualization of MAGL in the human brain [2]. [^18^F]-T-401-PET is considered a promising tool for measuring central MAGL. On the other hand, a first-in-human study using the ligand targeting the cannabinoid receptor has also been reported [3]. Endocannabinoid signaling stems from endocannabinoid binding to cannabinoid receptors, such as cannabinoid receptor type 1 (CB1R) and type 2 (CB2R). Recently, [^11^C]-MDTC, which has a high binding affinity for CB2R, was developed for PET imaging in vivo. [^11^C]-MDTC is a radioactive analog of A-936339, a selective CB2R agonist with a high affinity for CB2R. High CB2R expression in the brain tissue has been reported in neurological conditions, including postmortem cases of Alzheimer's disease (AD) and Parkinson's disease (PD). Imaging of CB2R using [^11^C]-MDTC may yield further insights into its role in shaping these neurological diseases, with the potential to inform and monitor relevant therapeutic interventions.

Neuroinflammation is also involved in the pathogenesis of neurodegenerative diseases, including AD and PD. In activated microglia and reactive astrocytes, the expression of the translocator protein 18 kDa (TSPO) is substantially upregulated, rendering it a valuable biomarker for neuroinflammation. TSPO may be a critical diagnostic target for all stages of AD, PD, and amyotrophic lateral sclerosis (ALS). Although various generations of TSPO PET ligands have been developed, their clinical utility is limited due to tracer sensitivity to the widespread rs6971 genetic polymorphism (A147T). Recently, several [^18^F]-labeled TSPO radioligands have been identified; among these, [^18^F]-LW223 is insensitive to the rs6971 polymorphism. In a first-in-human study, Tan et al. revealed that [^18^F]-LW223 effectively maps TSPO throughout the human brain and is suitable for quantitative TSPO-targeted PET imaging in a first-in-human study [4]. This novel imaging tool would enable noninvasive real-time quantification of TSPO, thus further improving our understanding of neuroinflammatory processes in the neurodegenerative brain.

In the last several years, anti-Aβ antibody treatment for AD has been implemented. Since amyloid PET has the potential to monitor the efficacy of anti-amyloid therapy and determine the indication for treatment, improving sensitive and specific A*β*-detection using PET is critical for earlier AD diagnosis and evaluation of therapeutic anti-A*β* drugs for AD. [^11^C]-Pittsburgh compound B ([^11^C]PiB), the first A*β*-specific PET radiotracer, is a benchmark probe for clinical A*β* diagnosis due to its high binding affinity and remarkable brain pharmacokinetics; however, the limitation of its short ^11^C radioactive decay half-life precludes its widespread use. As a result, ^18^F-labeled ligands such as [^18^F]-flutemetamol and [^18^F]-florbetapir are widely used in clinical and research settings. They have similar retention in A*β* deposits of AD patients as [^11^C]-PiB; however, all of them exhibit non-negligibly high non-specific binding in white matter. Considerable nonspecific white matter retention of tracers is an important limitation for early diagnosis and sensitive detection. In contrast, [^18^F]-92, the newly developed A*β*-specific PET ligand, displayed reliable binding to A*β* burden regions and little non-specific retention in white matter, crucial for distinguishing AD patients from cognitively healthy elders in the first-in-human study [5]. Therefore, [^18^F]-92 might become a promising PET tracer for visualizing A*β* pathology in AD patients. However, further studies and clinical applications are required in this regard.

On the other hand, tracers for imaging cerebral white matter are also essential in neurodegenerative diseases. Multiple sclerosis (MS) is the most common neurodegenerative disease in young adults. The disease process in MS involves inflammation, resulting in myelin loss, and possibly direct neurodegeneration. Some PET tracers of neuroinflammation have been tested in MS. However, none of these tracers selectively bind to myelin. Several tracers, originally developed for imaging A*β,* including [^11^C]-PiB, have been reported to bind to the beta-pleated sheet structure in myelin. Although [^11^C]-PiB uptake is only moderately correlated with myelin histology, [^11^C]-PiB PET detects demyelination in a primate model of MS. Animal studies have shown that [^11^C]-MeDAS may be more accurate for imaging myelin than [^11^C]-PiB. A first-in-man study of [^11^C]-MeDAS showed that this ligand could quantify myelin density in patients with MS and distinguish differences in myelin density within MS lesions [6]. [^18^F]-3F4AP has also shown promise for imaging demyelinated lesions in neurological diseases. [^18^F]-3F4AP is a radiofluorinated analog of the MS drug 4-amino-pyridine (dalfampridine, 4AP). Similar to 4AP, [^18^F]-3F4AP binds to voltage-gated K^+^ channels in demyelinated axons and has been proposed as a PET tracer for imaging demyelinated lesions in the brain. Brugarolas et al. first reported the biodistribution and radiation dosimetry of [^18^F]-3F4AP in humans for the first time [7]. Given that demyelination occurs in many diseases and is potentially reversible, tracers that allow quantitative imaging of demyelination in humans may contribute to a better understanding of the pathophysiological processes, a more accurate diagnosis of brain diseases, and provide a tool for accurately monitoring remyelinating therapies.

Huntington disease (HD) is an incurable neurological condition, wherein the mutant huntingtin (mHTT) protein accumulates in the brain and causes the disease, mainly via a toxic gain-of-function mechanism. Two new radioligands, [^11^C]-CHDI-00485180-R and [^11^C]-CHDI-00485626, were designed for the in vivo visualization of mHTT aggregates in the brain parenchyma of patients with HD. A first-in-human study indicated that [^11^C]-CHDI-00485180-R has promising kinetic properties in the brain, whereas [^11^C]-CHDI-00485626 is not suitable as a PET tracer for human brain mHTT aggregate quantification because of the possibility of radiometabolite accumulation in the brain parenchyma [8]. Current HD therapies are purely symptomatic and do not affect the disease course. Therefore, multiple therapeutic strategies are being developed to reduce the brain mHTT levels. PET ligands for mHTT would help evaluate the brain target engagement in mHTT-lowering clinical trials and could potentially serve as biomarkers for disease progression in HD.

Depression is a functional disorder that can occur in people of all generations, with its onset involving complex pathological processes and neurotransmitters. Phosphodiesterase-4 (PDE4) metabolizes the second messenger cyclic adenosine monophosphate (cAMP) and has four isozymes. Of these, PDE4B and PDE4E may play a role in the pathophysiology and treatment of depression and dementia. Therefore, PDE4 inhibitors are being explored as treatments for several neuropsychiatric disorders. [^18^F]-PF-06445974, a novel PET radioligand, was developed to preferentially bind to PDE4B. A first-in-human study on [^18^F]-PF-06445974 suggested that this radioligand quantified PDE4B reasonably [9]. If a PDE4B inhibitor is designed as an antidepressant, the PET radioligand can identify the appropriate dose and dosing interval for a therapeutic candidate. Additionally, a PDE4B radioligand may identify a subgroup of patients who are most likely to respond to a PDE4B inhibitor. The selection of patients who are likely to respond to these inhibitors is referred to as patient stratification, patient enrichment, and personalized medicine.

## Innovative tracers in oncology

The development of immune checkpoint inhibitors such as programmed death ligand 1 (PD-L1) inhibitors has revolutionized the management of advanced non-small cell lung cancer (NSCLC). However, based on limited biopsy samples, the current PD-L1 expression assessment via immunohistochemistry often fails to capture the heterogeneity of PD-L1 expression within and between tumors. Therefore, there is a need for PD-L1 PET imaging to enable the whole-body assessment of PD-L1. Although antibody-based tracers labeled with long-lived radionuclides (e.g., ^64^Cu and ^89^Zr) have shown promise, they have limitations, such as long blood circulation times, slow body clearance, and poor tumor penetration. To address these issues, tracers based on smaller molecules, single-domain antibodies, nanobodies, and small proteins, have been developed.

[^68^Ga]-NOTA-WL12 is a small peptide labeled with ^68^Ga, binding to PD-L1 with high affinity. Zhou et al. conducted the first-in-human study of [^68^Ga]-NOTA-WL12 in nine patients with advanced NSCLC [10]. [^68^Ga]-NOTA-WL12 with a tumor-to-lung ratio of 4.45 ± 1.89 at 1-h post-injection (p.i.). The SUVpeak of the tumors showed a strong correlation (r = 0.9349) with immunohistochemical PD-L1 expression assessed using the tumor proportion score (TPS). Furthermore, patients with positive uptake of [^68^Ga]-NOTA-WL12 responded better to pembrolizumab plus chemotherapy than those with negative uptake, indicating that patients with higher uptake might benefit from PD-L1 targeting immunotherapy. Another promising tracer, [^18^F]-NOTA-NF12 [11], exhibited predominant urinary excretion and lower nonspecific accumulation in the liver and small intestine compared to that with [^68^Ga]-NOTA-WL12. In the first-in-human trial by Zhou et al., which consisted of seven patients (NSCLC: *n* = 3; esophageal cancer: *n* = 4), patients with NSCLC with high PD-L1 expression had higher tumor uptake than those with low expression (SUVmax: 3.29 vs. 2.22). Similarly, esophageal cancer patients with moderate PD-L1 expression had higher uptake than those with low expression (SUVmax: 2.98 vs. 1.86). Ma et al. developed a ^68^Ga-labeled PD-L1-targeted nanobody PET tracer, [^68^Ga]-THP-APN09, which has the potential to be developed into a kit-based radiotracer because of its simple and mild labeling conditions [12]. In their first-in-human study, nine patients with resectable NSCLC underwent baseline [^68^Ga]-THP-APN09 PET/CT and subsequently received 2–4 cycles of neoadjuvant immunotherapy combined with chemotherapy, followed by surgery. The pathological response was evaluated and revealed a positive correlation (rs = 0.8763) between SUVmax and tumor immunohistochemical PD-L1 expression (TPS). Patients showing higher [^68^Ga]-THP-APN09 uptake in the tumor had a better response than non-responders (SUVmax 2.73 vs. 2.10), suggesting that the tumor uptake of [^68^Ga]-THP-APN09 may predict the benefit of neoadjuvant immunotherapy.

FDA-approved PSMA PET agents, such as [^68^Ga]-PSMA-11 and [^18^F]-DCFPyL, provide high diagnostic accuracy and are indispensable for the management of prostate cancer. However, further improvements are still required. For example, urinary excretion may hamper the identification of pelvic lesions. [^68^Ga]-P137 is an ODAP-urea-based PSMA ligand that enhances water solubility by replacing one carboxylate with a more hydrophilic oxalate in the ODAP-urea ligand, resulting in faster clearance from non-target organs. Duan et al. directly compared [^68^Ga]-P137 and [^68^Ga]-PSMA-617 PET/CT in three patients with prostate cancer who underwent both [^68^Ga]-P137 and [^68^Ga]-PSMA-617 PSMA PET within five days [13]. They reported that [^68^Ga]-P137 demonstrated much lower uptake in the urinary tract than [^68^Ga]-PSMA-617 (SUVmax ratio of [^68^Ga]-P137/[^68^Ga]-PSMA-617:0.20 ± 0.07) and that both tracers showed moderate to high uptake in prostate cancer lesions without a significant uptake difference between the two tracers.

Based on a similar concept is another PSMA agent, [^68^Ga]-PSMA-D5, which uses biphenyl as the core scaffold and exhibits less urinary excretion. Chen et al. compared [^68^Ga]-PSMA-D5 with [^68^Ga]-PSMA-617 in preclinical studies in mice [14]. [^68^Ga]-PSMA-D5 showed a higher tumor uptake (1.9-fold) and a higher tumor-to-kidney value (5.4-fold) than [^68^Ga]-PSMA-617, indicating rapid renal clearance. In their first-in-human study of two patients with prostate cancer, [^68^Ga]-PSMA-D5 effectively detected small prostate and metastatic lesions with high tumor-to-muscle (T/M) ratios (range: 56–73). In addition to the ^68^Ga-labeled agents, ^18^F-labeled PSMA agents are currently under development. Wu et al. reported promising results for Al[^18^F]-PSMA-Q, which introduced a quinoline ring to improve the hydrophilicity, tumor uptake, and clearance rates from normal organs and the blood pool [15]. In mice studies, compared to [^18^F]-DCFPyL, Al[^18^F]-PSMA-Q showed a shorter elimination half-life (DCFPyL vs. PSMA-Q: 14.22 vs. 3.93 min) and higher T/M ratio (13.25 vs.84.37). In a first-in-human study consisting of 26 tumor lesions in 20 patients with prostate cancer, Al[^18^F]-PSMA-Q had a T/M ratio of 44.77, which was higher than the previously reported T/M ratios of ^18^F-labeled PSMA agents, including [^18^F]-DCFPyL, [^18^F]-PSMA-1007, and [^18^F]-rh-PSMA-7. The radiation dose from a single Al[^18^F]-PSMA-Q PET/CT scan was calculated to be 0.0128 ± 0.007 mSv/MBq, which was lower than that reported for [^18^F]-DCFPyL (0.0165 mSv/MBq) and [^18^F]-PSMA-1007 (0.0220 mSv/MBq), possibly because of the faster renal excretion of Al[^18^F]-PSMA-Q.

The development of [^89^Zr]-labeled PSMA agents has also progressed. The longer half-life of ^89^Zr allows for prolonged storage and transportation, and its imaging capability may be extended to the detection of low PSMA-expressing tumors, which other short-life tracers may miss. Vázquez et al. reported the potential of [^89^Zr]-PSMA-DFO [16] and small-animal PET imaging demonstrated that tumor visualization with [^89^Zr]-PSMA-DFO was comparable to that of [^68^Ga]-PSMA-11 and [^18^F]-JK-PSMA-7 at early time points (1 h p.i.). In contrast, the tumor uptake of [^89^Zr]-PSMA-DFO PET increased at later time points (24 and 48 h p.i.), resulting in a higher T/M ratio than that of ^68^Ga- and ^18^F-labeled tracers. In its first-in-human application in a patient with prostate cancer and biochemical recurrence, [^89^Zr]-PSMA-DFO PET (48 h p.i.) showed local recurrence (SUVmax 13.25) that was equivocal (SUVmax 5.37) on [^18^F]-labeled PSMA PET ([^18^F]-JK-PSMA-7) at 2 h p.i. This indicates the potential advantage of [^89^Zr]-PSMA-DFO PET for localizing tumors with low PSMA expression.

Thyroid cancer (TC) is the most common endocrine malignancy. Nuclear medicine approaches primarily target the sodium iodide symporter (NIS), which is used for diagnosis and therapy. Recent advancements have highlighted new targets for the extracellular matrix protein fibronectin (FN) and its extra-domain B (EDB-FN), which are expressed in fetal and cancerous tissues, making them useful for targeted radioisotope delivery in cancers, including TC. EDB-FN-targeted peptides, named EDBp, have been applied in PET imaging with [^18^F]-NOTA-PEG4-EDBp and therapy with [^177^Lu]-DOTA-PEG4-EDBp ([^177^Lu]-EDBp). Initial human evaluations (n = 2) demonstrated the safety and specificity of [^18^F]-EDBp, whereas radionuclide therapy with [^177^Lu]-EDBp inhibited the tumor growth and prolonged survival in TC-bearing mice [17]. These findings lay the groundwork for further clinical applications in advanced TC management.

C-X-C motif chemokine receptor 4 (CXCR4) is necessary for tumor growth and metastasis, making it an attractive target in nuclear oncology. The CXCR4-targeting PET agent [^68^Ga]-Pentixafor has been administered to patients with various hematologic malignancies, allowing the identification of patients who can be treated with the therapeutic CXCR4-targeting radiotracer [^90^Y]/[^177^Lu]-Pentixafor. Considering the supply shortage of ^68^Ga, labeling with ^99m^Tc formulations offers a versatile alternative. The development of ^99m^Tc-PentixaTec provides an effective means, [^99m^Tc]-N_4_-dap-r-a-Abz-CPCR4 ([^99m^Tc]-N_4_-L6-CPCR4, ^99m^Tc-PentixaTec), for CXCR4 imaging, and the first human study was performed in five patients with hematological malignancies by Konrad et al. [18]. They demonstrated high uptake in CXCR4-expressing tissues, suitable clearance/biodistribution characteristics, and favorable human dosimetry. Thus, they have tremendous potential for further clinical use.

Human epidermal growth factor receptor type 2 (HER2) is a prominent molecular target for cancer therapy, particularly for ovarian, breast, gastric, and colorectal cancers. The PET tracer, [^18^F]-AlF-RESCA-MIRC213, showed rapid clearance and specific targeting of HER2-positive tumors in initial human studies (n = 6, breast cancer), making it a promising tool for noninvasive cancer diagnosis [19]. In the ten HER2-positive and -negative lesions, [^18^F]-FDG uptake was obvious, but the uptake of [^18^F]-AlF-RESCA-MIRC213 correlated well with the tumor HER2 expression level. Accurate differentiation of HER2 heterogeneity is crucial for evaluating the HER2 status and monitoring responses to HER2-targeted therapies.

c-MET is a receptor tyrosine kinase that is involved in various cellular processes, including the cancer cell growth, survival, and metastasis. Overexpression of c-MET is associated with poor prognosis in several types of cancer, including renal cell carcinoma (RCC). The PET tracer [^68^Ga]-EMP-100, a novel PET ligand based on a c-Met-binding peptide, was used to visualize c-MET expression in metastatic RCC and showed high uptake in primary tumors and metastases in the first human study (n = 12) [20]. Integrating this tracer into clinical practice, such as cabozantinib, could enhance the precision of cancer diagnosis and treatment planning by targeting c-METs.

Claudin 18 (CLDN18) is a member of the claudin protein family and is involved in the formation of cell-tight junctions, regulation of epithelial cell permeability, and prevention of diffusion of proteins and lipids on the cell membrane. CLDN18.2 is uniquely expressed in primary gastric cancer and its metastatic lesions as well as in pancreatic, esophageal, ovarian, lung, and colitis-associated colorectal tumors. Owing to its specific expression pattern, CLDN18.2 has become a potential target for treating epithelial tumors. Imaging with the CLDN18.2-targeted PET tracer [^124^I]-18B10(10 L) demonstrated high tracer uptake in metastatic lymph nodes, indicating its potential for detecting metastatic disease in a phase 0 trial for digestive system cancers, including 12 gastric cancers, four pancreatic cancers, and one cholangiocarcinoma (*n* = 17) [21]. This provides a non-invasive and effective molecular imaging method that can be used to screen patients and predict and evaluate the curative effects of CLDN18.2-targeted therapy.

Glutamine is a crucial nutrient for cellular growth and proliferation and plays a significant role in energy production, oxidative stress defense, and the biosynthesis of amino acids and nucleotides. The first-in-human study of the PET tracer L-[5-^11^C]-Glutamine in patients with metastatic colorectal cancer has shown specific and high uptake in tumor lesions, suggesting its potential as a non-invasive tool for cancer diagnosis and monitoring (n = 9) [22]. This approach could help in selecting appropriate therapies targeting the glutamine pathway and monitoring treatment responses, ultimately improving patient outcomes.

Ascorbic acid (Vitamin C) has been investigated for its role in cancer therapy, including its antioxidant properties, collagen synthesis, and immune system support. The PET tracer, 6-Deoxy-6-[^18^F]-L-ascorbic Acid ([^18^F]-DFA), an analog of ascorbic acid, has been used to assess ascorbic acid metabolism in various cancers. Long et al. reported a multivariate distribution pattern similar to that of ascorbic acid [23]. They displayed high uptake and retention in tumors with appropriate kinetics in the first human study, including kidney, scalp angiosarcoma, and colon, thyroid, and lung cancers (*n* = 6), suggesting its potential as a valuable tool for monitoring the distribution and therapeutic efficacy of ascorbic acid.

Metomidate is an inhibitor of the enzyme 11β-hydroxylase (CYP11B1) and aldosterone synthase (CYP11B2), which are found in the adrenal cortex, and ^11^C-labeled metomidate ([^11^C]MTO) is currently used for imaging of adrenocortical carcinoma and primary aldosteronism (PA) [24]. New tracers are being developed to improve the short half-lives and high hepatic accumulation of [^11^C]-MTO and [^18^F]-CETO (para-choloro-2-fluoroethyletomidate) is one of them. The first-in-human study of [^18^F]-CETO was performed in patients with various adrenal diseases and healthy volunteers [25]. The tracer showed high adrenal uptake and low hepatic accumulation, which improved assessment of the right adrenal gland. Normal adrenal uptake of [^18^F]-CETO is flow dependent, and further studies are required to investigate its uptake in adrenal diseases.

Macrophages expressing the macrophage mannose receptor (CD206) play a regulatory role in angiogenesis, invasion, and migration of cancer cells and are implicated in poor responses to therapy. Thus, CD206 is a potential target for tumor imaging. A first-in-human study of ^68^Ga-labeled anti-CD206 single-domain antibodies (sdAbs) was performed in patients with at least 10 mm solid tumors to evaluate their safety, biodistribution, and dosimetry [26]. The tracer was confirmed to be safe and the average effective dose was 4.2 mSv for males and 5.2 mSv for females. Blood clearance was rapid, with less than 20% of the radioactivity dose remaining after 80 min, and the tracer accumulated in organs expressing CD206, such as the liver, spleen, and bone marrow. Similar to other peptides and protein tracers, the tracer showed high accumulation in the kidneys. Three patients with high tracer accumulation in the tumor showed subsequent progression, whereas three patients with low tracer accumulation showed long-term progression-free survival at the post-imaging follow-up.

Imaging of cell death is of clinical value in assessing treatment responses in oncology. 4-(N-(S-glutathionylacetyl)amino) phenylarsonous acid (GSAO) is a tripeptide of trivalent arsenic. Radiolabeled GSAO is known to detect cell death by entering dying cells and binding to intracellular heat shock 90 proteins (HSP90). Thus, ^68^Ga-labeled GSAO (also referred to as Cell Death Indicator [CDI]) was prepared, and a first-in-human study was performed on patients with solid malignancy and at least one measurable lesion measuring > 2 cm [27]. [^68^Ga]-CDI is safe and its radiation dosimetry is similar to that of clinical ^68^Ga-labeled radiopharmaceuticals. Tracer uptake varied between tumors, which was speculated to be due to the varying proportion of dead cells resulting from necrosis in the tumor. Indeed, it showed high accumulation in tumors with many TUNEL-positive areas, a marker of apoptotic cell death. Thus, [^68^Ga]-CDI has the potential to improve treatment response assessments and enable cell death imaging.

To enhance tumor uptake and retention, Zang et al. designed a heterodimeric radiotracer targeting both fibroblast activation protein (FAP) and integrin α_v_β_3_ [28]. The quinoline-based FAP inhibitor (FAPI), FAPI-02, and the cyclic RGDfK peptide were combined using a PEG spacer and labeled with ^68^Ga to obtain [^68^Ga]-FAPI-RGD. The tracer showed higher tumor cell uptake in vitro and higher tumor accumulation and retention in tumor-bearing mice than its monomeric counterparts ([^68^Ga]-FAPI02 and [^68^Ga]-RGD). Subsequently, a clinical study was performed on patients with malignant tumors. The effective dose of [^68^Ga]-FAPI-RGD (1.94 mSv/100 MBq) was comparable to that of [^68^Ga]-FAPI tracers and [^68^Ga]-RGD tracers previously reported. The tracer showed rapid and high tumor uptake, long tumor retention, and high tumor-to-background ratios and was mainly excreted from the urinary system.

## Innovative tracers: miscellaneous

This first-in-human study was performed on diseases other than neurological and oncological diseases. Wall et al. performed PET imaging of amyloidosis [29], which is characterized by the deposition of amyloid proteins in the body, and early diagnosis can improve patient outcomes. In preclinical studies, the radiolabeled peptide p5 + 14, an amyloid-binding peptide consisting of 45 amino acid residues, could image systemic amyloids in a murine model and showed colocalization with Congo red-positive deposits in all tissues [30]. The first-in-human study of ^124^I-labeled p5 + 14 was performed in three patients with systemic amyloidosis. The estimated half-life of the tracer in blood was 21.9 ± 7.6 h. The tracer rapidly accumulated in the organs in a patient-dependent manner, suggesting specific retention of amyloid deposits in each patient. As a result of dehalogenation of the tracer, radioactivity was also observed in the stomach, parotid, salivary, and thyroid glands. In contrast to patients with amyloidosis, radioactivity was observed only in the organs where free iodine accumulated in healthy subjects.

Beckford-Vera et al. attempted the PET imaging of human immunodeficiency virus (HIV) infections [31]. The prognosis of patients infected with HIV has improved with the advent of antiretroviral therapies (ART). However, HIV largely resides outside the peripheral circulation, making it difficult to understand the tissue-wide burden of HIV infection and its anatomical distribution using routine sampling. ^89^Zr-labeled broadly neutralizing antibody against HIV, [^89^Zr]-VRC01, was prepared, and a first-in-human study was performed on five viremic patients, five ART-suppressed patients, and five uninfected control participants. [^89^Zr]-VRC01 uptake significantly increased in various lymphoid and other tissues from patients with HIV infection. In addition, tracer uptake in the inguinal lymph nodes of patients significantly and positively correlated with intracellular HIV protein expression of sampling lymph nodes. These results indicate that [^89^Zr]-VRC01 has the potential for imaging residual HIV infection.

In summary, several novel tracers were developed, of which some can be routinely used in the future. In the clinical setting, availability, affordability, and diagnostic performance are significant factors for broad acceptance. Progress in medical instruments is an essential aspect of the utility of these new radiopharmaceuticals. Although some tracers may disappear before they are used clinically, continuing to develop novel tracers can brighten the future of this field.
